# Stress and the Commensal Microbiota: Importance in Parturition and Infant Neurodevelopment

**DOI:** 10.3389/fpsyt.2015.00005

**Published:** 2015-02-02

**Authors:** Tamar L. Gur, Brett L. Worly, Michael T. Bailey

**Affiliations:** ^1^Psychiatry and Behavioral Health, The Ohio State University Wexner Medical Center, Columbus, OH, USA; ^2^Department of Obstetrics and Gynecology, The Ohio State University Wexner Medical Center, Columbus, OH, USA; ^3^Department of Neuroscience, The Ohio State University Wexner Medical Center, Columbus, OH, USA; ^4^Division of Biosciences, College of Dentistry, The Ohio State University, Columbus, OH, USA; ^5^Institute for Behavioral Medicine Research, The Ohio State University Wexner Medical Center, Columbus, OH, USA; ^6^Department of Pediatrics, The Ohio State University Wexner Medical Center, Columbus, OH, USA; ^7^Comprehensive Cancer Center, The Ohio State University Wexner Medical Center, Columbus, OH, USA

**Keywords:** microbiota, psychological stress, neurodevelopment, prematurity, neuroimmune, anxiety, depression

## Abstract

The body is colonized by an enormous array of microbes that are collectively called the microbiota. During quiescent periods, microbial communities within the gut are relatively resistant to change. However, several factors that disrupt homeostasis can also significantly change gut microbial community structure. One factor that has been shown to change the composition of the gut microbiota is exposure to psychological stressors. Studies demonstrate that the commensal microbiota are involved in stressor-induced immunomodulation, but other biological effects are not yet known. This review discusses emerging evidence that the microbiota can impact the brain and behavior and indicates that stressor-induced alterations in the composition of gut microbial communities contribute to stressor-induced behavioral changes. This review will also discuss the evidence that such effects are most evident early in life, where both stress and the microbiota have been linked to birth outcomes, such as prematurity, and neurodevelopment. When considered together, a paradigm emerges in which stressor-induced alterations in commensal microbial populations significantly impact parturition and infant neurodevelopment.

## Introduction

**Figure 1:**
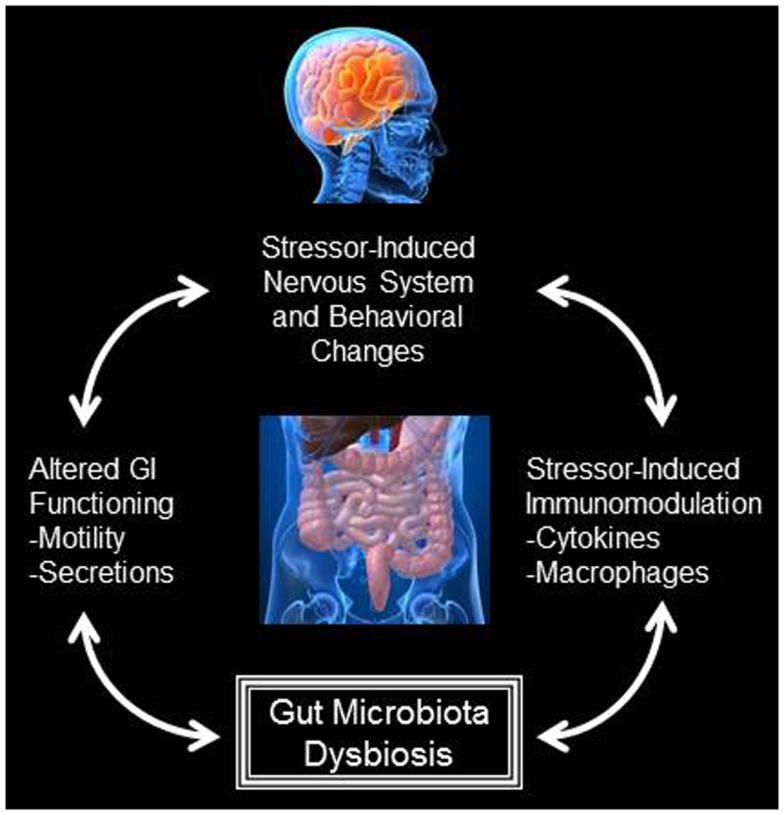
**Bidirectional interactions exist between the central nervous system and the gut microbiota**. During exposure to stressful stimuli, the physiological stress response can lead to alterations in gastrointestinal functioning, such as alterations to gastrointestinal motility and the secretion of factors like gastric acid and bile. The physiological stress response is also known to impact immune system activity. Both alterations to gastrointestinal functioning and immune system activity can significantly change the composition of the gut microbiota. These alterations to the microbiota can feedback and impact gastrointestinal functioning, immune system activity, as well as the physiological stress response and stressor-induced behavioral states.

The hypothesis that the origins of adult disease are developmental, beginning *in utero* is called the “Barker hypothesis” after one of its leading proponents and the author of a study demonstrating increased risk of cardiovascular disease in infants born underweight (1). It states that adverse influences early in development, such as poor nutrition or infection, result in permanent physiological changes and in increased disease risk in adulthood. This is an area of increasing research in many fields, including Psychiatry, Immunology, and Endocrinology, which are now exploring whether development of diseases such as asthma, diabetes, and anxiety contains a developmental, intrauterine element. Neurodevelopment is exquisitely sensitive to perturbations in the maternal milieu, including diet, infection, and stress, with potentially long-lasting behavioral consequences. Disorders such as schizophrenia, anxiety, depression, and autism have been found to be associated with *in utero* and early neonatal exposure to these stimuli (2). Infants exposed to antenatal stress demonstrate increased risk of developing a host of childhood and adult diseases. While alterations in the hypothalamic–pituitary–adrenal (HPA) axis and immune function have been the target of investigation as underlying mechanisms conferring increased risk, the microbiome is an emerging candidate as a potential mediator of stress-induced pathogenesis (Figure 1).

Every surface of the body naturally harbors unique microbial communities comprised of archea, protists, viruses, and bacteria. To date, bacteria residing on mucosal surfaces, including the oral cavity, reproductive tract, and gastrointestinal tract, are the best characterized. These bacteria form highly ordered microbial communities as a result of ecological successions that select microbes that are best adapted for their given niche. Although these microbial communities are relatively resistant to change, it is recognized that factors such as alterations in diet and the administration of antibiotics can result in modifications in microbial community structure. Studies from this laboratory, as well as others, have demonstrated that psychosocial stressors can also impact microbial community structure in the gut. This review will briefly describe studies that have linked stressor-induced alterations in gut microbial community structure to alterations in immune system activity and behavioral responses. The potential impact of these interactions on pregnancy outcome and on infant development will also be discussed.

## Neuroendocrine-Bacterial Interactions

The field of psychoneuroimmunology has amply demonstrated that the physiological response to different types of stressor significantly impacts immune system reactivity to antigenic challenge [reviewed in Ref. (3)]. Primary mediators of the stress response, including endogenous glucocorticoids such as cortisol in humans and corticosterone in rodents, can affect immune system reactivity by suppressing the expression and activity of key transcription factors, such as NF-kB (4). Likewise, stressor-induced activation of the sympathetic nervous system, resulting in the release of endogenous catecholamine hormones (namely epinephrine from the adrenal medulla, and norepinephrine from adrenergic nerve terminals), can significantly increase or decrease immune cell activity depending on the leukocyte subset and the adrenergic receptor that is bound. As details of the importance of neuroendocrine mediators for leukocyte reactivity emerged (5), findings demonstrating that bacterial pathogens themselves also respond to neuroendocrine hormones began to emerge (6–12).

The growth of many types of bacteria, including both infectious and commensal organisms can be significantly impacted by neuroendocrine hormones. For example, the growth of commensal and of pathogenic *E. coli* can be increased over 10,000-fold by simply adding norepinephrine to a serum-based microbial medium (8, 10, 12–14). It is now recognized that a wide variety of neuroendocrine hormones can impact a vast array of bacteria in culture [reviewed in Ref. (10, 13)], however, demonstrating that direct neuroendocrine–bacterial interactions occur *in vivo* has been more challenging.

One of the first studies assessing the effects of neuroendocrine hormones on bacterial growth involved increasing norepinephrine levels *in vivo* using 6-hydroxydopamine, which lyses sympathetic nerve terminals. This resulted in an approximately 10,000-fold increase in commensal *E. coli* levels in the cecum of mice (11). The effects of norepinephrine on bacterial growth was also evident in an ileal loop model, where growing *Salmonella enterica* with norepinephrine prior to inoculation into the ileal loop significantly increased pathogen growth and associated disease (15). Findings that neuroendocrine mediators associated with the stress response could significantly impact bacterial growth led us to test whether stressor exposure could significantly change the levels of bacteria cultured from the intestines.

## Stressor Exposure and the Commensal Microbiota

Tannock and Savage demonstrated almost 40 years ago that moving mice into a cage lacking bedding, food, and water reduced the number of lactobacilli that could be cultured from the gastrointestinal tract (16). While this suggested that the stress response associated with new housing led to the differences, it was difficult to interpret these data due to the food and water deprivation. Thus, infant rhesus monkeys, with *ad libitum* access to food and water were exposed to a maternal separation stressor and the number of lactobacilli that could be cultured from the intestines was assessed. Exposure to the stressor significantly reduced lactobacilli levels, and the magnitude of the reduction was associated with stress-indicative behaviors. In general, monkeys which showed the greatest behavioral signs of distress also had the lowest levels of lactobacilli (17).

Stressor-induced reductions in lactobacilli have also been identified in humans during the stress of school examinations (18) and in murine studies utilizing prolonged restraint (19) or a short-lasting social stressor (19). The biological importance of stressor-induced alterations of the microbiota is not well understood. However, studies demonstrate that some aspects of stressor-induced increases in immune system reactivity are dependent upon the microbiota. For example, exposure to a social stressor, known as social disruption (SDR), significantly increases IL-6 levels in the blood and increases the reactivity of splenic macrophages to microbial stimulation. These effects, however, did not appear in germ-free (GF) mice or mice treated with antibiotics to reduce the microbiota (20). Similarly, exposing rats to repeat tail shock significantly increases cytokine levels in the blood; treatment with antibiotics to reduce the microbiota attenuated stressor-induced increases, but only for cytokines whose activation is dependent upon the inflammasome (21, 22). When considered together, these studies demonstrate the importance of the microbiota for stressor-induced immunopotentiation, and suggest that microbial products act as a danger signal to prime the immune system for enhanced reactivity (23).

## Stress, the Microbiome, and Preterm Birth

**Figure 2:**
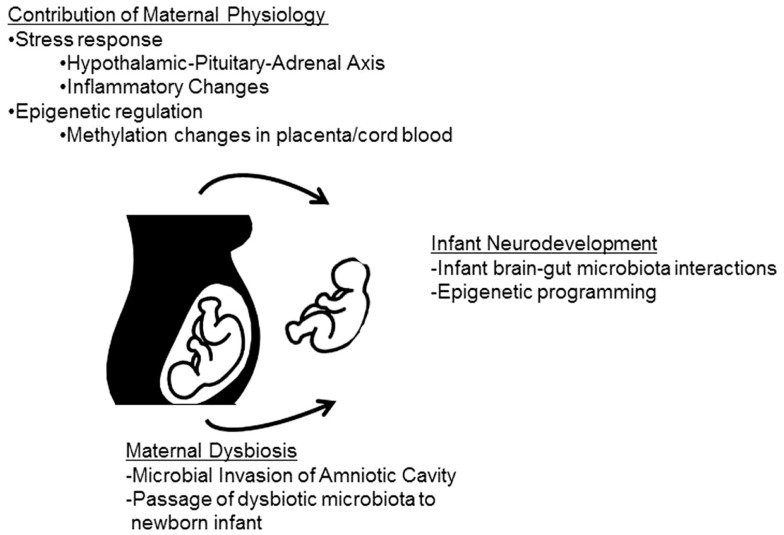
**Maternal stress can impact infant neurodevelopment through both microbiota-dependent and -independent pathways**. The maternal physiological stress response can directly impact fetal development and may be involved with epigenetic changes in the offspring. In addition, the maternal physiological stress response may impact the commensal microbiota. Infants may become colonized with altered maternal microbes either *in utero*, through bacterial invasion of the amniotic cavity, or during/after birth when maternal microbes are passed to the infant. Either alterations in infant physiology (including epigenetic modifications) or colonization with dysbiotic microbial communities may lead to deficiencies in infant neurodevelopment.

While it is becoming increasing evident that stressor-induced alterations in the microbiota of adult animals can significantly impact host physiology, these effects are transient and return to baseline after termination of the stressor. However, the microbiota have exaggerated and prolonged effects when perturbed during gestation or early in infancy. Interest in the connection between stress and the human microbiome, and its impact specifically on female reproduction and the developing fetus is evolving, but still in a nascent stage (Figure 2).

Preterm birth (PTB) is the first critical juncture to look at in the reproductive cycle when trying to discern the effects of stress and the microbiome on women, as stress is a well-known risk factor that affects the pillars of PTB: immune response/inflammation, and the HPA axis (24). There is considerable debate about stress, inflammation, cortisol, and PTB, and exact mechanisms are ripe for evaluation. Microbial invasion of the amniotic cavity and the associated host inflammatory response is a leading etiology of PTB (25–28). The mechanisms by which micro-organisms gain access to the decidua and amniotic cavity are not completely known, but are thought to involve invasion from microbes locally (e.g., lower gut or genital tract) or hematogenously (25). The ability of genital pathogens to invade the amnion and induce PTB has been well studied. However, the source of hematogenous microbes and their ability to invade the amniotic cavity remains unclear. Oral microbes have been proposed as a key source of microbes passed to the amniotic cavity via hematogenous transmission (29). This is based on recent evidence that the placenta harbors a commensal microbiome with a community structure that is more closely reflective of oral microbial communities than microbial communities found elsewhere in the body (30). In addition, periodontal diseases are often, but not always (31), related to increased risk of PTB (32). It is thought that during some cases of periodontal infection, oral pathogens, such as *Fusobacterium nucleatum* translocate from the oral cavity into circulation where they eventually reach and invade the pregnant uterus (32). Given the strong association between stress and PTB, as well as oral microbiota and PTB, it is important to consider the possibility that oral microbes play a critical role in linking the stress response to PTB. While it is known that stressor exposure can change the composition of the gut microbiota, and the ability of microbes to translocate from the lumen of the intestines to the interior of the body (19, 33–35), to date, it is not known whether stressor exposure also impacts the composition of the oral microbiota, or their ability to translocate to other organs. Moreover, the relationship between the various commensal microbial communities and the intrauterine environment is not well understood in part due to the lack of studies involving laboratory animal models, and this is certainly an important area of future studies.

## Stress, the Microbiome, and Parturition

With information suggesting that early-life exposures can be formative for neurodevelopmental, allergic, and immunological response later in life, attention can be turned to parturition for potential influencing factors specifically with the microbiome. Two studies suggest that the neonatal fecal microbiome is altered due to cesarean section at day 1 of life, and continues up to 6 weeks of age (36, 37). Meta-analysis suggests that a 20% increase in asthma rates exists among babies born with cesarean section, although substantial heterogeneity exists in the data (38). Breastfed babies have a different, more “beneficial” gut microbiota (39, 40), obtain different prebiotic human milk oligosaccharides (41), and acquire maternal intestinal bacteria from breast milk (42). In addition to having different routes of delivery, it is interesting to note that babies born via cesarean section are also born to mothers with higher levels of stress. Cesarean section, especially unplanned cesarean section, is perceived as severely stressful (43, 44) and can lead to significant increases in glucorticoid and catecholamine neuroendocrine hormones (45, 46). Thus, while the mode of delivery is undoubtedly important, delivery via cesarean section may have additional effects on the baby due to the high physiological stress response in the mother. For example, cesarean section is associated with a different human milk microbiome, potentially as a result of the stress of cesarean section. While never described before for stress, it is recognized that overall health of the mother can impact the milk microbiome; obese women’s breast milk microbiome has been shown to be less diverse than healthy weight controls (47). Thus, it is likely that stress and hormonal alterations impact breast milk microbiome composition. Interestingly, early changes in the human gut microbiome with perinatal antibiotics were found to have an association with increased disease risks early in life (48).

## Neonatal Microbiome and Neurodevelopment

There is accumulating evidence that the microbiome can influence behavior [Reviewed in Ref. (49)], supporting the concept of a microbiome–gut–brain axis. While specific mechanisms underlying this influence remain unknown, the presence of the microbiome in the placenta and amnion suggest proximity and capacity to influence the developing fetus. Studies examining the impact of microbiome on the developing central nervous system (CNS) have utilized GF mice that have decreased anxiety, as well as increased motor activity compared to conventional mice. These behavioral changes were accompanied by increased turnover of dopamine, norepinephrine, and serotonin in the striatum, though not, it should be noted, in the hippocampus or frontal cortex. Levels of brain-derived neurotrophic factor (BDNF), a protein with a significant role in neurodevelopment, and the development of depression and anxiety, were found to be reduced in the hippocampus and amygdala. These are brain regions implicated in the pathogenesis of anxiety, suggesting a mechanistic substrate for the behavioral changes (50). Because BDNF has a significant role in neurodevelopment, these findings have tantalizing implications regarding the influence of stress, alterations in the microbiome, neonatal colonization, and neurodevelopmental disorders.

Not all aspects of neurodevelopment are influenced by the microbiota, and some components of microbial influences on neurodevelopment are gender-specific. For example, GF mice have also demonstrated significant deficits in social behavior (51), some of which, specifically social avoidance, were reversed with re-colonization post-weaning. However, social cognition did not improve with re-colonization, suggesting that social cognition is as amenable to microbial-based interventions.

The effects that the microbiota have on the developing CNS appear to be gender-dependent. GF male mice have increased levels of serotonin and its metabolites in their hippocampus, and these did not normalize with introduction of a regular microbiome following weaning. Immunological and neuroendocrine effects were also found, with GF mice demonstrating a blunted immune response, based on TNF-α production, with a larger effect in female mice. GF animals also had a stronger corticosterone response in relation to stress, with female mice showing a smaller response. Male GF mice showed decreased expression of BDNF; however, female mice did not (52).

When considered together, data from laboratory animals indicate that maternal microbes that are passed to the infant are necessary for normal neurodevelopment. Disrupting these microbial communities, or their passage to the infant, can in turn impact neurodevelopment. Moreover, there are significant sex differences in the impact of microbial colonization on neurodevelopment. While it is not yet clear how this occurs, sex difference mechanisms in mice may be due to estrous cycle hormones and the CNS serotonergic system, as the estrogen receptor (ER beta) has a role in the hippocampal serotonin concentration. It is unclear whether the estrogen and estrogen receptor have a larger impact, or whether the microbiome has a larger impact on serotonin, and further studies are needed.

## Neonatal Microbiome and Health Outcomes

Animal studies support the idea that alterations in neonatal life and gut microbiome can have substantial impact on long-lasting health. Rat offspring deprived of their mother showed increased permeability of the colonic mucosa and a 10- to 100-fold increase in bacterial adherence to colonic tissue and spleen translocation. Of note, these changes were prevented by injecting rat pups with a corticotropin-releasing hormone receptor antagonist daily during maternal separation, suggesting that the HPA axis mediated this effect (53). Further evidence of maternal influence on the offspring is provided by the finding that, in rats, maternal diet influenced offspring gut architecture (54) and high-fat diet in rat mothers led to decreases in maltase and sucrase in offspring, while *E. coli* introduced to rat mothers in pregnancy and lactation led to increased offspring intestinal permeability, systemic inflammation, and obesity for this next generation (55). Furthermore, *E coli* introduced in rat mothers led to changes in offspring weight and gut microbiota (56). Maternal administration of antibiotics in the prenatal period was associated with increased gut permeability and systemic inflammation (54) as well as increased visceral sensitivity (57). In rodents, maternal separation, a highly validated model of early-life stress, also lead to an increase in visceral sensitivity, with concomitant alterations in stress response and microbial community structure (58). A swine model for perinatal disturbance was adopted with oral antibiotics to sows. Short- and long-term changes were seen in paracellular permeability (59). Together, these studies suggest that altering either the maternal or perinatal flora has long-lasting implications on the gut of the offspring, as well as immunological repercussions. Mechanisms for this change are still being evaluated, but one potential route is that perinatal changes in bacterial colonization alter gastrointestinal heat shock protein expression with permanent implications on health (60).

## How the Microbiome may Impact Neurodevelopment and Future Directions

While clear lines of evidence exist supporting an effect of stress on the microbiome, as well as the ability of stress to modulate neurodevelopment and behavioral changes, a delineated mechanism of action by the microbiome upon the CNS is an area of intense scrutiny and investigation (61–63). To date, the HPA axis and alterations in the immune response have accumulated the strongest evidence of associations with alterations in the microbiome.

The immune system, in addition to being directly influenced by stress, is a direct target of both microbiota and probiotic agents, and also has bidirectional communication with the CNS, making it an appealing candidate. The microbiome, through the innate immune system, alters levels of both pro- and anti-inflammatory cytokines, which are capable of directly impacting CNS function (64, 65). Stress also has a clear impact on the HPA axis, which is capable of regulating the inflammatory cascade, and growth of microbiota, as discussed above. However, directly tethering this phenomenon to the developing CNS is a more arduous task. While studies reviewed above demonstrate alterations in important players in CNS development, such as BDNF, with alterations (or lack of) microbiota, many steps in this process remain elusive.

Another potential mediator of the effect of stress on neurodevelopment, via the microbiome, is epigenetic regulation (66). This refers to heritable changes in gene expression not due to changes in DNA sequence, and epigenetic modification is a process known to be especially sensitive to early-life experiences. Thus, *in utero* and early-life alterations in microbiome could potentially utilize epigenetic processes to exert long-term behavioral changes, such as those described following psychosocial stressors. Indeed, a growing body of literature supports a major role of epigenetic modification in the neurobiology of psychiatric disorders (67). How might the microbiome influence epigenetics? Microbiotas are involved in the breakdown of nutrients, and in that process create metabolites with neuroactive properties, including amino acids and monoamines (68). Moreover, they are a key source of butyrate, which is a histone deacetylase (HDAC) inhibitor, a key step in transcriptional regulation. However, whether these substances are able to make a substantial impact on CNS function itself, and how they would access the CNS from the periphery, is open to debate and requires further examination.

As a whole, the ability of the microbiome to impact the developing CNS, and participate in the effect of psychosocial stress is an exciting concept, because it is susceptible to targeting with pre-and probiotics, which has vast implications for both neurodevelopment and other health outcomes. While much work remains to be done to elucidate mechanisms, this daunting task is imperative to complete.

## Conflict of Interest Statement

The authors declare that the research was conducted in the absence of any commercial or financial relationships that could be construed as a potential conflict of interest.
